# A model-agnostic framework for dataset-specific selection of missing value imputation methods in pain-related numerical data

**DOI:** 10.1080/24740527.2025.2595160

**Published:** 2026-01-29

**Authors:** Jörn Lötsch, Alfred Ultsch

**Affiliations:** a Institute of Clinical Pharmacology, Goethe – University, Frankfurt am Main, Germany; b Faculty of Medicine, University of Helsinki, Helsinki, Finland; c Fraunhofer Institute for Translational Medicine and Pharmacology ITMP, Frankfurt am Main, Germany; d DataBionics Research Group, University of Marburg, Marburg, Germany

**Keywords:** Data science, data preprocessing, pain-related data, bioinformatics, pain thresholds, opioid analgescis

## Abstract

Missing value imputation is a routine step in biomedical data analysis, yet techniques are often not tailored to specific datasets. We propose a systematic framework for selecting imputation methods customized for the unique characteristics of cross-sectional numerical data, with a focus on pain-related biomedical research. This approach generates artificial “diagnostic” missing values by randomly removing entries, allowing for direct assessment of reconstruction accuracy across various algorithms. We introduce two novel classes of diagnostic reference methods: pseudo or “poisoned” imputation methods, which intentionally introduce bias into the imputation, and “calibrating” imputations, which inject controlled random noise for objective evaluation. The framework was tested on synthetic datasets and four biomedical datasets, primarily focusing on pain-related data, employing 29 different imputation methods. Quantitative outputs, including root median square deviation (RMSD), median difference (MD), relative bias, and method categorization, facilitate a comprehensive assessment of imputation quality. The framework consistently identifies the most suitable imputation technique for each dataset, revealing that multivariate methods generally outperform univariate approaches. Benchmarking against poisoned and calibrated references establishes quantifiable thresholds for acceptable imputation errors, while also identifying instances where reliable imputations are unattainable. This systematic framework offers practical and reproducible guidelines for imputing missing values in biomedical contexts, particularly in pain research. By empowering researchers to make informed decisions about imputation, the framework enhances data integrity and the robustness of subsequent analyses. Its model-agnostic nature allows for the integration of various imputation methods, with an automated implementation available in the open-source R package “opImputation.”

## RÉSUMÉ

L’imputation des valeurs manquantes est une étape courante dans l’analyse des données biomédicales, mais il arrive souvent que les techniques ne soient pas adaptées à des ensembles de données spécifiques. Nous proposons un cadre systématique pour la sélection de méthodes d’imputation personnalisées en fonction des caractéristiques propres aux données numériques transversales, en mettant l’accent sur la recherche biomédicale liée à la douleur. Cette approche génère des valeurs manquantes artificielles « diagnostiques » en supprimant aléatoirement des valeurs, ce qui permet une évaluation directe de l’exactitude de la reconstruction au moyen de divers algorithmes. Nous introduisons deux nouvelles classes de méthodes de référence diagnostiques : des méthodes d’imputation pseudo ou « empoisonnées », qui intègrent intentionnellement un biais dans l’imputation, et des méthodes d’imputation « calibrées », qui injectent un bruit aléatoire contrôlé afin de permettre une évaluation objective. Le cadre a été testé sur des ensembles de données synthétiques ainsi que sur quatre ensembles de données biomédicales, principalement liées à la douleur, à l’aide de 29 méthodes d’imputation différentes. Les résultats quantitatifs, incluant la racine de l’écart quadratique médian, la différence médiane, le biais relatif et la catégorisation des méthodes, permettent une évaluation complète de la qualité de l’imputation. Le cadre identifie de manière cohérente la technique d’imputation la plus appropriée pour chaque ensemble de données, montrant que les méthodes multivariées surpassent généralement les approches univariées. La comparaison aux références empoisonnées et calibrées permet d’établir des seuils quantifiables d’erreur d’imputation, tout en repérant les situations où il n’est pas possible d’obtenir une imputation fiable. Ce cadre systématique offre des lignes directrices pratiques et reproductibles pour l’imputation des valeurs manquantes en contexte biomédical, en particulier dans la recherche sur la douleur. En donnant aux chercheurs les moyens de prendre des décisions éclairées en matière d’imputation, le cadre améliore l’intégrité des données et la robustesse des analyses ultérieures. Son caractère indépendant du modèle permet l’intégration de différentes méthodes d’imputation, et une mise en œuvre automatisée est offerte dans le progiciel R libre « opImputation ».

## Introduction

Missing value imputation is a critical step in biomedical data analysis, since incomplete datasets can introduce bias and reduce statistical power if not properly addressed.^1^^2^ Pain research is no exception, and incomplete numerical tabular datasets are often encountered. While some traditional statistical analyses can still run with partial data, requiring only precise reporting of the degrees of freedom, methods such as machine learning typically demand complete data. Researchers may restrict analyses to complete cases, but this quickly reduces sample size to levels where reliable results cannot be obtained. Alternatively, missing values can be replaced with estimated entries.

The choice of imputation method, however, is often arbitrary. Standard statistical software packages provide only limited options, such as substituting missing values with the median or mean, or applying a small selection of multivariate approaches, such as regression-based linear trend imputation, iterative Markov chain Monte Carlo (MCMC),^3^ or predictive mean matching (PMM).^4^ More recently, machine learning-based techniques implemented in code-based environments have offered a wider range of options and often outperformed classical methods.^5^ Yet selecting the best method for a given dataset remains difficult. General recommendations exist but lack specificity, and the large variety of algorithms underlines the absence of universally applicable guidelines.^1 6^

Thus, the decision on which imputation method to use should be based on diagnostics of the dataset under investigation rather than on generic comparative benchmarks of various imputation methods.^6^ To address this, we propose a systematic approach for dataset-specific comparison of multiple imputation strategies. Our method introduces artificial “diagnostic” missing values by randomly deleting a proportion of existing entries, enabling direct measurement of how accurately each algorithm reconstructs the original data. The present approach introduces two novel classes of diagnostic reference methods crucial for evaluating imputation strategies. Pseudo (or “poisoned”) imputations are designed to intentionally produce biased or inaccurate results, establishing a clear negative benchmark for comparison. In contrast, calibrated imputations replace missing values with the true value plus controlled random noise, allowing for a quantitative assessment of reconstruction accuracy. These benchmarks facilitate a meaningful interpretation of actual imputation algorithms by providing performance standards against known reference points. Ultimately, this framework aims to offer clear guidance on selecting either a single imputation method or a tailored set of methods for the specific dataset being analyzed.

## Methods

The proposed method evaluates multiple imputation algorithms to identify the most appropriate option(s) for a given dataset. The focus is on numerical, cross‑sectional, tabular data, where observations are arranged in rows and columns and measured at a single point in time.^7^ In contrast to domains such as time‑series analysis of brain function or pharmacokinetics, where imputation strategies are explicitly tailored to temporal models or field‑specific standards (e.g., handling concentrations beyond limits of quantification as non‑random), cross‑sectional datasets often lack such conventions. The guiding premise here is that the optimal method should restore missing values with maximum accuracy. Rather than aiming for a universal ranking, our approach provides practical, reproducible, and data‑specific recommendations. The intent is an efficient screening process that outperforms arbitrary method selection, while still allowing researchers the flexibility to apply more advanced techniques if desired.

### Framework design

#### Creation of artificial missing values

All evaluations were based on initially complete datasets, into which artificial missingness was introduced by systematically masking selected entries.^6 7^ Two complementary patterns were simulated:

- Persistent missingness: A fixed subset of entries was removed at the start with probability *p* (e.g., 0.1), mimicking stable dropout patterns common in biomedical data. These positions remained missing throughout all experiments.
- Diagnostic missingness: In each iteration, a new random subset of entries was removed and restored after the cycle. This ensured that the true diagnostic values were always known, enabling direct, iteration-wise evaluation of imputation accuracy.^6^

Comparisons of imputation methods were based solely on diagnostic values, reflecting practical use where the true entries can serve as a reference. Persistent missingness, in contrast, was retained to assess the final, automatically selected imputation method. This separation parallels the validation–test split used in machine learning.

The guiding principle is that performance on artificially generated gaps generalizes to real missing values. The objective of any imputation is to recover values that are as close as possible to the true entries, while avoiding systematic errors such as consistent over- or underestimation. Following established practices for comparing measurement methods,^8 9^ this principle is formalized into evaluation metrics that quantify both accuracy and bias.

#### Imputation quality metrics

To quantify performance, three complementary measures are applied and later combined into one measure.

##### Error magnitude

The root median square deviation (RMSD) quantifies the overall error magnitude:

(1) $$\mathrm{RMSD}\left(y_{i}^{\prime },y_{i}\right)=\sqrt{\mathrm{median}\left({\left(y_{i}^{\prime }-y_{i}\right)}^{2}\right)}$$

where $y_{i}^{\prime }$ are the imputed values of a variable and $y_{i}$ are the true values for each artificially missing value in variable i. The median is used as a robust estimator due to the typical non-normality of differences in biomedical data.^8^

##### Absolute bias

The median absolute difference (MD) detects consistent over‑ or underestimation:

(2) $$\mathrm{MD}\left(y_{i}^{\prime },y_{i}\right)=\left|\mathrm{median}\left(y_{i}^{\prime }-y_{i}\right)\right|$$

This measures systematic bias, that is, if imputed values are consistently higher or lower than the true values.

##### Relative bias

Value‑dependent error is assessed via the regression slope (*b*_1_) from a robust (rank-based) linear regression of the difference between imputed and true values against their mean, as suggested by Bland and Altman:^9^

(3) $$\mathrm{rBias}\left(y_{i}^{\prime },y_{i}\right)=\left\{\begin{array}{cc} \left|\frac{\left(y_{i}^{\prime }-y_{i}\right)-b_{0}}{\bar{y_{i}^{\prime },y_{i}}}\right| & \mathrm{if}\bar{y_{i}^{\prime },y_{i}}\;\neq \;0\\ 0 & \mathrm{otherwise} \end{array}\right.$$

This captures trends where the imputation error varies with the value magnitude.

All three metrics are directed toward lower values, with an optimal imputation technique achieving values of zero for each. Since these metrics are sensitive to scale,^10^ their ranks are used for comparison, such that the lowest (best) value is ranked first.

To prevent negligible differences from dominating rankings, each metric is adjusted using a Wilcoxon test.^11 12^ If deviation from zero is not statistically significant (*p* ≥ 0.1), the metric is set to zero for ranking:

(4) $${\bar{\mathrm{metric}}}_{i,j}=\left\{\begin{array}{cc} \mathrm{metric}\left(y_{i}^{\prime },y_{i}\right) & \mathrm{if}\;p_{t}<0.1\\ 0 & \mathrm{otherwise} \end{array}\right.$$

where $\mathrm{metric}\left(y_{i}^{\prime },y_{i}\right)$ is one of the metrics defined above at the $j^{\mathrm{th}}$ repeated measure for the variable with $p_{t}$ being the *p*-value obtained from a non-parametric test.

#### Ranking and aggregation of imputation methods

Imputation methods are ranked for each modified measure and variable. Let V be the number of variables, and the number of repeated trials. The overall ranking for a specific imputation method, considering all metrics, is:

(5) $$R_{m}=\frac{1}{V\cdot k}\overset{V}{\underset{i=1}{\sum }}\overset{k}{\underset{j=1}{\sum }}\frac{\left({\bar{\mathrm{RMSE}}}_{i,j}\;+\;{\bar{\mathrm{MD}}}_{i,j}\;+\;{\bar{\mathrm{rBias}}}_{i,j}\right)}{3}$$

meaning the average score over all metrics, variables, and repeated trials.

The best imputation method is the one that minimizes the average rank:

(6) $$\mathrm{Best}\;\mathrm{Imputation}\;\mathrm{Method}=\frac{a\mathrm{rgmin}\left(R_{m}\right)}{\mathrm{m\varepsilon}1\ldots M}$$

#### Dataset-specific top method selection (Via cABC)

Since several algorithms may perform similarly well, instead of selecting only a single “winner,” we apply computed ABC (cABC) analysis^13^ to define three groups: important few (A), acceptable (B), and trivial many (C)

The ranking distribution (*R_i_*) of imputation methods is modeled as a special case of the Irwin-Hall distribution for uniformly distributed ranks.^14 15^ For large numbers of variables (V) and trials (*k*), the central limit theorem allows approximation by a Gaussian distribution $N\left(m,s\right)$, where *m* = (*M* + 1)/2 and $s=M/\sqrt{12d}$, with d=V⋅k and M the number of methods.^16^

The standardized average rank is:

(7) $$Z_{R}\left(i\right)=\frac{R_{i}-m}{s}$$

The *p*-value for each algorithm is then:

(8) $$p\left(i\right)=\mathrm{cdf}\left(Z_{R}\left(i\right),m,s\right)$$

where *cdf* is the cumulative distribution function. The cABC value is:

(9) $$\mathrm{ABC}\left(i\right)=\left\{\begin{array}{c} Z_{R}{\left(i\right)}^{2}\;\mathrm{for}\;Z_{R}\left(i\right)<0\\ 0\;\mathrm{otherwise} \end{array}\right.$$

Algorithms in the cABC subset A are considered the best for the given dataset.

#### Scale-independent accuracy

As an additional evaluation, the mean absolute difference between standardized (*z*‑scored) true and imputed values is reported:

(10) $$\mathrm{\Delta z}=\frac{1}{n}\sum_{i=1}^{n}\left|Z_{\mathrm{imputed},i}-Z_{\mathrm{orig},i}\right|$$

where n is the number of artificially missing values, and $Z_{\mathrm{orig}}$ and $Z_{\mathrm{imputed}}$ are the *z*-scores of the original and imputed values, respectively:

(11) $$Z_{\mathrm{orig}}=\frac{\mathrm{orig}-m}{s},\;Z_{\mathrm{imputed}}=\frac{\mathrm{imputed}-m}{s}$$

with m and s being the mean and standard deviation of the complete data for the variable. In figures, this value is denoted as “zDelta”. This “zDelta” quantifies imputation accuracy independently of variable scale.

### Benchmarking strategy

Two categories of benchmarking methods were incorporated, providing reference points for expected performance.

#### Negative benchmarks (“poisoned” methods)

These methods deliberately generate biased values, named by analogy to “poisoned” data, as described in.^17^ If no genuine imputation method outperforms them, the conclusion is that imputation is not advisable for that dataset. In our study, we used three variants of such poisoned methods.

##### Absolute bias (PLUS)

A constant value is added to the true value for each missing entry:

(12) $$\mathrm{PLUS}:\mathrm{yij}=y_{\mathrm{ij}}+c^{\;}.E\left(\left|y_{j}\right|\right)\mathrm{ifE}\left(\left|y_{j}\right|\right)\neq 01\mathrm{otherwise}$$

where c≠0 and the median is used as a robust estimator.

##### Relative bias (FACTOR)

Each true value is multiplied by a constant:

(13) $$\mathrm{FACTOR}:\mathrm{yij}=y_{\mathrm{ij}}+c^{.}E\left(\left|y_{j}\right|\right)\mathrm{ifE}\left(\left|y_{j}\right|\right)\neq 01\mathrm{otherwise}$$

with c≠0.

##### Alternating bias (PLUSMINUS)

Positive and negative constants are alternately added to successive values:

(14) $$\mathrm{PLUSMINUS}:y_{ij}^{\prime }=y_{\mathrm{ij}}+{\left(-1\right)}^{i}\cdot \left[1+c\cdot \left\{\begin{array}{c} E\left(\left|y_{j}\right|\right)\;\mathrm{if}\;E\left(\left|y_{j}\right|\right)\;\neq \;0\\ 1\;\mathrm{otherwise} \end{array}\right.\right]$$

with c≠0. This method does not introduce directional bias, as the expected value remains unchanged.

#### Quantitative benchmarks (“calibrating” methods)

These replace missing values with the true value plus controlled random noise. By varying the noise level (*c*), they provide a performance scale for situating real methods.

(15) $$\mathrm{tinyNois}e_{0.000001\ldots 1}:y_{ij}^{\prime }=y_{\mathrm{ij}}+U\left(n,\pm c\cdot \left\{\begin{array}{c} E\left(\left|y_{j}\right|\right)\;\mathrm{if}\;E\left(\left|y_{j}\right|\right)\;\neq \;0\\ 1\;\mathrm{otherwise} \end{array}\right.\right)$$

where $U\left(n\pm c\right)$ is a random value in $\left[-c,+c\right]$ and c is varied across a range (e.g., $\left[0.000001,0.00001,0.0001,0.001,0.01,0.05,0.1,0.2,0.5,1\right]$).

Together, poisoned and calibrating methods define meaningful lower and graded reference points. True imputation algorithms must outperform the poisoned controls and ideally match or exceed low-noise calibrating methods. If performance falls short, imputation is not recommended for the dataset.

### Evaluation datasets

The evaluation of the proposed approach employed both synthetic benchmark datasets and real biomedical datasets. Three synthetic three‑dimensional datasets were constructed to pose specific challenges for imputation and to facilitate transparent evaluation, as their structure is fully defined and mathematically controlled. Such benchmark simulations are widely used in the development and comparison of statistical algorithms, as they permit rigorous testing under known conditions before application to real data (e.g.,).^18 19^ Detailed results for the synthetic datasets are provided in the Supplementary Materials, with selected examples shown here for illustration. Complementing these benchmarks, we applied the method to complete biomedical datasets, primarily from pain research, in which missing values were introduced to mimic realistic patterns. Multiple imputation algorithms were then evaluated in a structured, iterative framework, and we further examined the impact of automated method selection on downstream analyses such as clustering.

The following sections describe: (i) the datasets used, (ii) the selection of imputation algorithms, (iii) the imputation protocol, and (iv) the analytical workflow for evaluating and comparing imputation strategies.

#### Synthetic data

To establish controlled benchmarks for method evaluation, we first employed three synthetic three‑dimensional datasets. Their fully defined and mathematically controlled structure allows transparent testing and makes it possible to study specific challenges of imputation under known conditions. The first dataset, “TwoLinearXY,” forms an X‑shaped pattern of two intersecting linear relationships, designed as a setting where multivariate imputation methods can exploit clear dependencies among variables. The second dataset, “UniformRandom3VarIndependent,” consists of three independent uniformly distributed variables representing pure noise, where multivariate approaches are not expected to outperform univariate methods.

The third dataset, “FCPSHepta,”^20^ consists of seven well-separated clusters embedded in three-dimensional space. This dataset was used to investigate the impact of missing value treatment on downstream analyses, such as clustering, serving as a basis for discussion. Full details on the generation and properties of this dataset are provided in the Supplementary Materials.

#### Biomedical datasets with a (non-exclusive) pain focus

##### Dataset #1 (“QSTpainEJPtransf”)

This psychophysical dataset originates from a clinical quantitative sensory testing (QST) experiment involving 127 healthy subjects (72 with complete data: 34 men, 38 women).^21^ It includes 19 pain measures: 9 from classical pain models (e.g., pressure, cold, electric, chemical, and laser-evoked pain thresholds and intensities), and 10 from a clinically established QST battery.^22 23^ The QST parameters encompass a range of thermal and mechanical pain and sensation thresholds. Measures were harmonized to ensure higher values indicate greater pain, and log-transformed as appropriate. Detailed protocols and variable descriptions are available in the original publication. Ethical approval and informed consent were obtained (see declarations at the end of this report).

##### Dataset #2 (“PainThresholds”)

Derived from the same research project, this dataset includes pain thresholds to various stimuli in 125 unrelated healthy volunteers (69 men, 56 women, aged 18–46).^24 25^ It comprises eight variables measuring sensory thresholds to the following stimuli: heat (“Heat”), cold (“Cold”), blunt pressure (“Pressure”), punctate pressure with von Frey filaments (“von.Frey”), electrical stimuli (“Electric”), heat after capsaicin sensitization (“Heat.Caps”), von Frey after capsaicin sensitization (“von.Frey.Caps”), and cold pain after menthol sensitization (“Cold.Menth”). The data form a 125 × 8 numerical matrix. Ethical approval and participant consent were as above.

##### Dataset #3 (“CodeinLogMetabolitesUrine”)

This pharmacogenetic dataset assesses codeine metabolism in 50 healthy subjects, measuring urine concentrations of codeine and four metabolites (C6G, morphine, M3G, M6G) by mass spectrometry.^26^ The dataset is complete, with no missing values. Ethical approval and informed consent were obtained.

##### Dataset #4 (“LipidsPsychiatricPat”)

This lipidomics dataset comprises blood concentrations of eight lipid mediators (S1P, C16Sphinganin, C16Cer, C20Cer, C24Cer, C24_1Cer, C16 GluCer, C16LacCer) measured in 212 samples. This is a subset of a larger dataset (see below), selected based on prior studies identifying these lipids as informative for psychiatric diagnosis.^27 28^ It did not contain missing values, so the assessments of inserted missing values could be performed as with the other datasets. Ethical approval and informed consent were obtained.

The full original lipidomics dataset from the referenced study^27 28^ was also used in its complete version, comprising 35 lipid mediators, including but not limited to AEA, ATP, cAMP, and multiple ceramides and lysophosphatidic acids (dataset #4b). This dataset underwent transformation and outlier removal, but retained 18 true missing values prior to imputation. It was employed to investigate the impact of missing value treatment on downstream analyses, such as classification, serving as an additional point of discussion.

#### Selection of imputation algorithms

The evaluation considered a broad range of established imputation methods,^6^ grouped into two categories:

- Univariate imputation methods use only information within the same variable (e.g., mean, median, mode replacement, or random resampling from available values).
- Multivariate imputation methods exploit relationships across variables, including distance‑based (k‑nearest neighbors,^29^) predictive mean matching (PMM),^4 30^ linear regression, and tree‑based models such as classification and regression tree (CART),^31^ bagged trees,^32–34^ and random forests.^35 36^ Multiple imputation by chained equations (MICE),^37 38^ and implementations from the R packages Amelia^39^ and mi^40^ were also included.

Special attention was given to the “missForest” R package use,^41^ which uses repeated random forest regressions for each variable with missing values, iteratively predicting them from the remaining variables. Unlike standard machine learning workflows, no explicit training/test split is made; all observed values serve as predictors, and missing values are prediction targets.

Machine learning methods introduce variability due to stochastic optimization. To ensure reproducibility, a fixed random seed was set. For key stochastic methods (RF, PMM, CART, bagged trees, and MICE variants), results were averaged from 20 repeated imputations, increasing computational demand, but reducing data noise in the imputations.

### Analysis workflow

**Figure 1.**
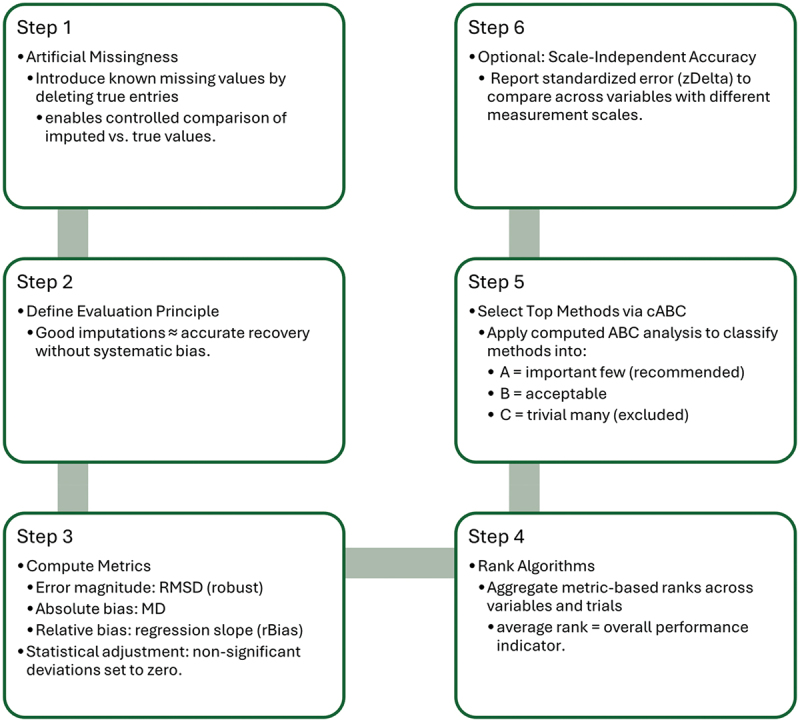
Sequential steps for rigorous imputation method comparison are illustrated. Artificial missingness is introduced by deleting known values, enabling controlled assessment of imputation accuracy. Evaluation is guided by principles of accurate recovery without systematic bias and quantified using robust error metrics (RMSD, median difference, and regression-based relative bias), with statistical adjustment for non-significant deviations. Algorithms are ranked by aggregated performance across variables and iterations. Top candidate methods are selected using cABC analysis, which classifies models by their importance and reliability. Optionally, standardized error (Δz) is reported to facilitate comparison across heterogeneous variable scales.

Evaluation started with complete datasets that were preprocessed as required (e.g., normalization or transformation), a step left to the researcher and not embedded in the imputation algorithms. Artificial missing values were then introduced in both persistent and diagnostic patterns, as illustrated in Figure 1. Each imputation algorithm was applied to these datasets, with replication for stochastic methods.

Accuracy was quantified on diagnostic entries, whose true values were known, using root mean square deviation (RMSD), median difference (MD), and relative bias (rBias). Derived from these evaluations, the primary quantitative output, Δz, specifically measures the standardized deviation between original and imputed values, serving as a vital metric for assessing the effectiveness of the imputation methods. Nonsignificant deviations were set to zero to avoid inflating differences. Results were aggregated across variables and repetitions, and methods were ranked by average performance.

Benchmarks included intentionally biased pseudo-methods and calibrating noise methods to determine whether meaningful imputation was possible for the given dataset. A computed ABC (cABC) analysis then classified algorithms into recommended (A), acceptable (B), and negligible (C) groups, providing dataset-specific recommendations. Finally, selected methods were tested on persistent missing values, and their impact on downstream analyses was assessed (Figure 1)

### Implementation

#### Software and package design

The imputation method selection framework was developed in the R language^42^ using the R statistical environment^43^ (version 4.3.3) and the PyCharm IDE (Professional Edition, version 2025.2.3, JetBrains s.r.o., Prague, Czech Republic), which offers an AI Assistant plugin (https://plugins.jetbrains.com/plugin/22282jetbrainsaiassistant) to support code generation and documentation (version 252.26830.157). The resulting R package, “opImputation,” provides functions for performing competitive imputation analyses, comparing methods, and generating visual and numerical summaries. The package is available at https://github.com/JornLotsch/opImputation (submitted to the Comprehensive R Archive Network, CRAN; publication there pending).

#### Functions and usage overview

The model-agnostic framework is implemented as the open-source package “opImputation.” Its primary function, compare_imputation_methods(…), accepts a numeric data frame or matrix to perform automated benchmarking of multiple imputation algorithms. Input data are validated to confirm a strictly numeric tabular structure; if no missing values are detected, the function halts and returns the original dataset as the imputed result, thereby avoiding unnecessary computations.

For datasets with missing values, the function carries out repeated imputation and evaluation cycles, governed by user-defined parameters, including the number of repetitions, diagnostic iterations, random seed, proportion of missing data, and number of processor cores for parallel computing. The parallelization utilizes R’s parallel package and seamlessly scales across Windows and Unix-like systems. Missingness mechanisms can be simulated as missing completely at random (MCAR) or missing not at random (MNAR), with adjustable degree and shape parameters. Supported algorithms encompass univariate methods (mean, median), multivariate techniques (k-nearest neighbors, regression, random forest), and multiple imputation approaches. After evaluating all algorithms, the framework can automatically select and apply the best-performing method to produce the final imputed dataset, serving both as an evaluation platform and an automated imputation engine. A detailed README elucidating argument descriptions and workflows is available in the GitHub repository.

#### Quantitative output and result reporting

**Figure 2.**
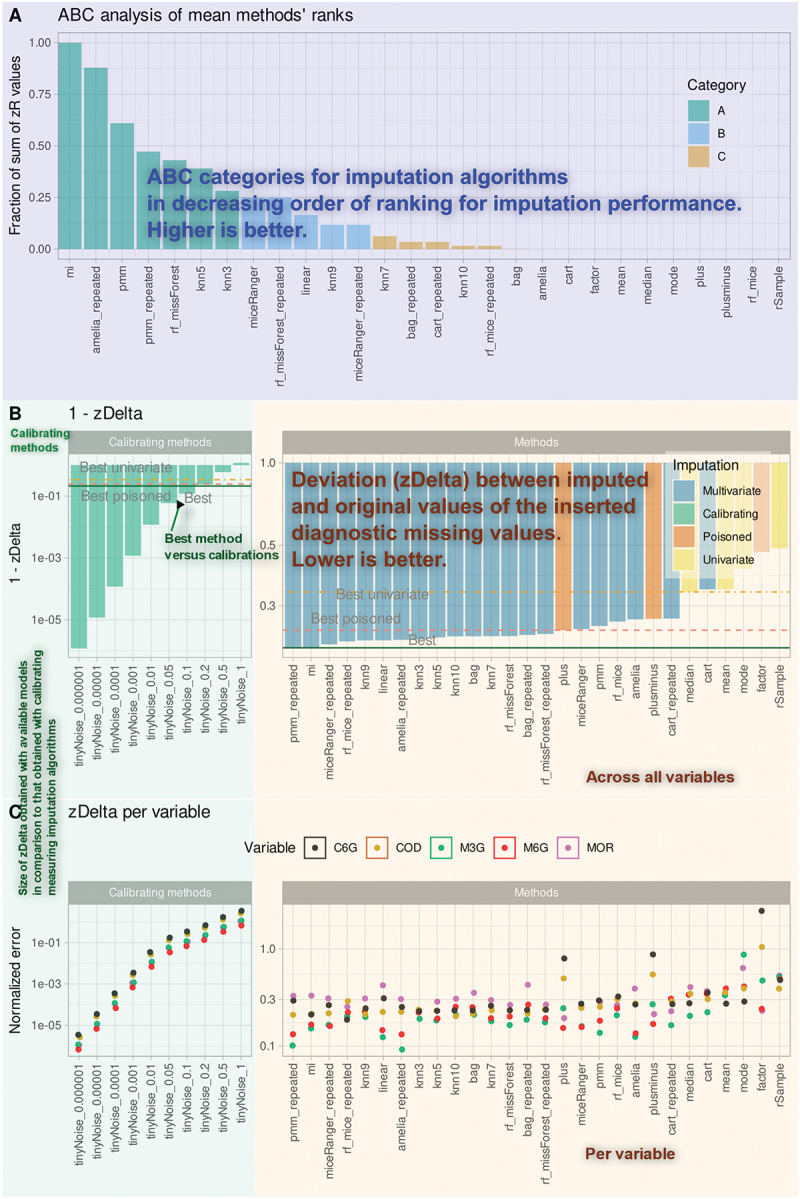
Annotated summary of results from comparative analyses of expected imputation results from different multivariate, univariate, and diagnostic algorithms. The analyses were performed exemplarily in dataset #3 (“CodeinLogMetabolitesUrine”). A: Bar graph of standardized mean ranks of imputation methods, color-coded by cABC categories and scaled to fit ABC curves. ABC sets A, B, and C represent best, next best, and discouraged models. The superimposed ABC curve (black line) shows the increasing proportion of the total sum of the *z*-transformed mean ranks of the imputation models from bottom to top, and the increasing proportion of the total ranks from left to right. B: Bar charts showing the mean absolute standardized errors, zΔ, obtained with different imputation models using inserted “diagnostic” missing values. The color code represents univariate (yellow), multivariate (blue), or pseudo (“poisoned,” orange) imputation models. Horizontal lines mark the best models within each model type. An “x” denotes an early termination of the imputation with an error message, which is occasionally observed. The left panel shows the zΔ values obtained with the calibrated imputation models. C: Mean values of zΔ per variable and imputation model over all imputation iterations. The figure was generated using R software (version 4.3.3 for Linux; https://CRAN.R-project.org/)^44^ and the “ggplot2” library (https://cran.r-project.org/package=ggplot2).^45^.

The framework generates reproducible quantitative output detailing imputation performance across all tested methods and variables. The central performance metric, Δz (z delta), measures the standardized, scale-independent deviation between the original and imputed values, forming the basis of the activity-based classification (ABC) analysis. Aggregated ABC metrics yield ranked tables of methods accompanied by corresponding scores and categories, identifying top-performing algorithms classified within the “A class,” which are presented in tabular and graphical formats (e.g., Figure 2A).

Structured numeric results are complemented by ggplot2-based summary figures that integrate ABC classifications with overall and variable-specific Δz distributions. When “produce_final_imputations = TRUE,” the output includes the fully imputed dataset, the selected algorithm, and the corresponding ABC statistics. Alternatively, the impute_data(…) function can be utilized to generate the final imputed dataset. An example of function syntax and installation procedures is provided in the Supplementary Textbox 1. All elements are returned in a single structured list suitable for reproducible workflows. For each dataset, the tool reports:

- The top-performing imputation methods
- An estimate of expected imputation accuracy (Figure 1B)
- A comparison between multivariate and univariate (e.g., median) imputation techniques (Figure 2D,E)
- Diagnostic signals indicating if none of the available methods yield satisfactory imputation (Supplementary Figure 2B,E)
- Warnings regarding the potential consequences of poor imputation on downstream analyses (Supplementary Figure 2C)
- Textual and numeric output, with an example displayed in Supplementary Table 1

**Textbox 1:** Model-agnostic pseudocode of the comparative framework for imputation evaluation

Step	Description
Input	D = {X₁, …, X_j_}, complete dataset with variables X_j_; set of imputation methods M = {m₁, …, m_m_}; persistent missingness probability p; number of repetitions k
1	Create artificial missing values
2	Randomly mask entries in D with probability p to generate persistent missingness (kept fixed across runs).
3	For each iteration j _i_ {1, …, k}:
3a	Randomly mask a new subset of entries to simulate diagnostic missingness.
3b	Save original values of masked entries for evaluation.
4	Initialize evaluation metrics for each variable i and method m: RMSD_ij_, MD_ij_, rBias_ij_.
5	Evaluate imputation methods
5a	For each method m _m_ M:
5b	For each repetition j = 1, …, k:
5b.i	Apply m to impute missing entries in D.
5b.ii	Compute RMSD: √(median((y_i_ y_i_)²))
5b.iii	Compute MD: |median(y_i_′− y_i_)|
5b.iv	Compute rBias via robust regression slope b₁ between imputed–true differences and their means.
5b.v	Perform Wilcoxon test; if p ≥ 0.1, set metric to zero.
5b.vi	Store adjusted metrics RMSD_ij_, MD_ij_, rias_ij_.
6	Aggregate results into overall ranking for each method m:
	R_m_ = (1 / (V·k)) Σ_i=_₁^V^ Σ_j=_₁^K^ [(MSD_ij_ + MD_ij_ + rias_ij_) / 3]
7	Select top imputation methods using cABC analysis
7a	Model rank distribution R_i_ as Irwin–Hall distribution: mean m = (M + 1)/2; variance s = M / √(12·d), where d = V·k
7b	Compute standardized score: Z^R^_i_ = (R_i_ − m) / s
7c	Compute p-value: p(i) = cdf(Z_j_, m, s)
7d	Determine cABC value: ABC(i) = {Z^R^_i_² for Z < 0; 0 otherwise}
7e	Define subset A (top-ranked algorithms) as those with lowest ABC values.
8	Compute scale-independent accuracy (optional)
8a	Standardize true and imputed values: Z_ori_g = (orig − m) / s; Z_imp_ = (imputed − m) / s
8b	Compute mean absolute difference: Δz = (1/n) Σ_i=_₁^n^ |Z_imp,j_ − Z_ori_g,_i_|
8c	Report as “zDelta”.
9	Include benchmarking controls
9a	Apply poisoned baselines (PLUS, FACTOR, PLUSMINUS) to verify performance gap.
9b	Apply calibrating reference models (tinyNoise_c) over predefined levels of c.
10	Return ranked imputation methods R_m_, identified top subsets (A, B, C), and scale-independent accuracies (zDelta).

### Experimentation

Analyses were performed on an Intel Core i7-13700 H notebook running Ubuntu Linux 22.04.4 LTS. A range of imputation algorithms was evaluated (Table 1), with implementations sourced from both standard R code and established R packages. Bagged CART imputation was performed using the “caret” package (https://cran.r-project.org/package=caret).^46^ Random forest-based imputation was carried out using three libraries—“mice,” “missForest” (https://cran.r-project.org/package=missForest),^41^ and “miceRanger” (https://cran.r-project.org/package=miceRanger)^47^—to account for differences in numerical results and error handling. In cases of failed imputation, missing values were retained as NA, allowing the selection process to exclude unsuccessful methods. Linear, CART, and PMM imputations were performed using the “mice” package (https://cran.r-project.org/package = mice).^48^ K-nearest neighbor imputation was included from the “multiUS” package (https://cran.r-project.org/package=multiUS)^49^ with fixed k values (3, 5, 7, 9, and the package default of 10). Multiple imputation approaches were also integrated from the “Amelia” (https://cran.r-project.org/package=Amelia)^39^ and “mi” (https://cran.r-project.org/package=mi)^40^ packages.

**Table 1.** Implemented imputation methods and their origin in R packages.

Name	Method	Originating R package
ameliaImp	Multiple imputation	“Amelia”
ameliaImp_repeated	As above, expected value from 20 imputation repetitions with different random start values	
bag	Bagged classification and regression tre3s	“caret”
bag_repeated	See above	
cart	classification and regression tress	“mice”
cart_repeated		
factor	Poisoned method	Actual
knn3	k-nearest neighbors, *k* = 3	“multiUS”
knn5	k-nearest neighbors, *k* = 5	
knn7	k-nearest neighbors, *k* = 7	
knn9	k-nearest neighbors, *k* = 9	
knn10	k-nearest neighbors, *k* = 10	
linear	Linear imputation	“mice”
mean	Mean	Actual
median	Median	
miceRanger	Random forests	“miceRanger”
miceRanger_repeated	See above	
maim	Multiple imputation	“mi”
mode	Mode	Actual
plus	Poisoned method	
plusminus	Poisoned method	
pmm	Predictive mean matching	“mice”
pmm_repeated	See above	
rf_mice	Random forests	
rf_mice_repeated	See above	
rf_missForest	Random forests	“missForest”
rf_missForest_repeated	See above	
rSample	Random value from variable	Actual
tinyNoise_0.000001	Calibrating method	
tinyNoise_0.00001		
tinyNoise_0.0001		
tinyNoise_0.001		
tinyNoise_0.01		
tinyNoise_0.05		
tinyNoise_0.1		
tinyNoise_0.2		
tinyNoise_0.5		
tinyNoise_1		

Computed ABC analyses were performed using “ABCanalysis” (https://cran.r-project.org/package=ABCanalysis).^13^ To compare the statistical performance of the best multivariate and univariate methods, Δz values were assessed using the DTS test from the “twosamples” package (https://cran.r-project.org/package=twosamples),^50^ complemented by a two-sample Wilcoxon signed rank test.^11^ Statistical significance was determined by combining results from both tests using Fisher’s method.^51^

## Results

All datasets were first assessed for distributional properties and, where appropriate, transformed (e.g., log-transformation) to enhance comparability and suitability for imputation. These preprocessing steps were completed prior to introducing missing values and are the responsibility of the researcher.

### Imputation performance and method ranking

**Figure 3.**
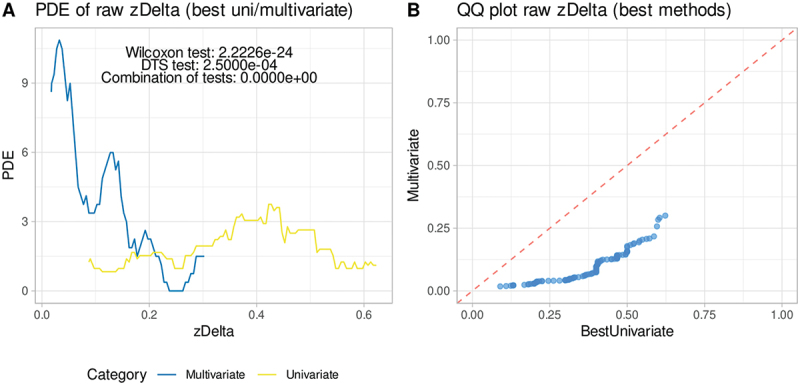
Distributions of zΔ values observed with the selected highest-ranking imputation model versus the respective best univariate or multivariate model (dataset #3 (“CodeinLogMetabolitesUrine”). A: Density distribution plotted using Pareto density estimation (PDE.)^52^ Statistical test results are annotated at the top. B: Quantile-quantile plot of zΔ values observed with the selected top-ranked imputation model versus the respective best univariate or multivariate model. The dashed line indicates identity. The greater the distance of the points from this line, the better one or the other type of imputation is in the dataset. The figure was generated using R software (version 4.3.3 for Linux; https://CRAN.R-project.org/)^44^ and the “ggplot2” library (https://cran.r-project.org/package=ggplot2).^45^.

Across all biomedical and synthetic datasets, multivariate imputation methods (Figure 2) consistently outperformed univariate approaches. For example, in dataset #3 (“CodeinLogMetabolitesUrine”), multivariate methods dominated the cABC “A” subset of top-performing models (Figure 2A), while univariate and “poisoned” methods were relegated to the irrelevant “C” subset. Multiple imputations (“mi”) achieved superior accuracy, consistently maintaining errors below a 20 percent margin when benchmarked against calibrating methods that add defined noise (Figure 2B). These results were robust across individual variables and overall means, with statistical analyses (e.g., *p*-values) confirming the superiority of multivariate imputation (Figure 3).

Within the same dataset (“CodeinLogMetabolitesUrine”), substituting missing values with the mean or median led to higher error rates compared to machine-learned values from other variables (Figure 2C). Quantile-quantile plots (Figure 3), a standard visualization tool for biomedical data, further illustrated the inferiority of univariate methods, emphasizing the enhanced efficacy of multivariate imputation.

### Benchmarking against pseudo and calibrating methods

**Figure 4.**
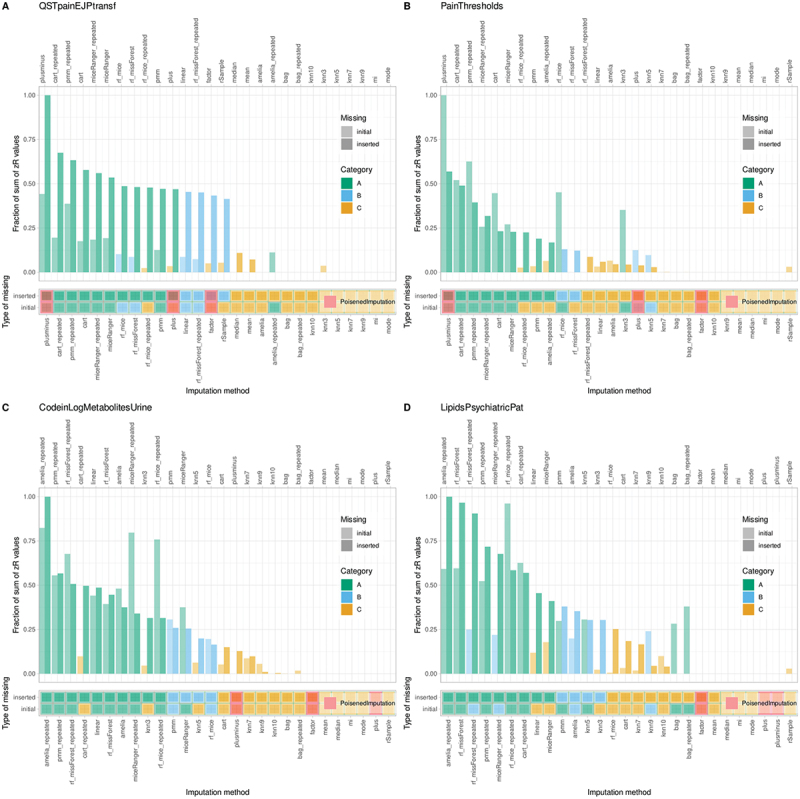
Item categorization of imputation models based on standardized ranks from the proposed imputation metric, examining imputation models across three performance measures, variables within the dataset, and iterations with randomly inserted diagnostic missing values. Four biomedical datasets from pain-related topics (psychophysical, pharmacological, lipidomics) are analyzed (datasets #1 through #4). Top subpanels: Bar graphs of standardized mean ranks, color-coded by cABC categories of the imputation algorithm, and scaled to fit ABC curves. ABC sets A, B, and C represent best, next best, and discouraged models. The superimposed ABC curves (gray lines) show from bottom to top the increasing fraction of the total sum of *z*-transformed mean ranks of the imputation models, and from left to right the increasing fraction of the total ranks. Boundaries are indicated by dotted or dashed lines. Bottom subpanels: Mosaic plot showing the membership of imputation models to cABC-based categories of imputation models. Red marks indicate “poisoned” models, which should be ranked last, otherwise problems with imputation of the dataset by the available methods are signaled. The figure was generated using R software (version 4.3.3 for Linux; https://CRAN.R-project.org/)^44^ and the “ggplot2” library (https://cran.r-project.org/package=ggplot2).^45^.

To contextualize imputation performance, the study applied intentionally “poisoned” and calibrating methods. For the pain sensitivity datasets (#1 “QSTpainEJPtransf” and #2 “PainThresholds”), a poisoned method was ranked first (Figure 4), highlighting challenges in imputability and supporting previous decisions to restrict analysis to complete cases. The artificially introduced calibrating methods, which added increasing levels of noise, provided a spectrum of expected errors. The best “true” imputation methods performed between the lowest and moderate noise thresholds, validating the benchmarking framework.

By contrast, for the other biomedical datasets (#3 “CodeinLogMetabolitesUrine” #4, “LipidsPsychiatricPat”), clear winning imputation methods were identified. These methods consistently outperformed both poisoned and calibrating approaches, providing tangible decision support for how to best impute these datasets. Multivariate techniques remained dominant, reinforcing the central findings that robust underlying data structure enables reliable imputation and meaningful comparative ranking of algorithms.

These patterns were also observed in artificial datasets. In structured data (e.g., dataset #5), multivariate methods outperformed both univariate and poisoned approaches, with “mi” ranking highest and yielding errors below 5 percent (Supplementary Figure 1, left). Poisoned methods reliably occupied the cABC “C” subset. In contrast, for dataset #6 (containing only random independent variables), poisoned methods were ranked in subset “A” and outperformed all others, indicating non-imputability (Supplementary Figure 1, right).

### Generalizability of diagnostic to true missing values

Competitive evaluation based on artificially introduced diagnostic missing values reliably identified methods that also performed best on original (persistent) missing values. In every dataset, the top method for diagnostic missing values was among the cABC “A” subset for true missing values (Figure 4), supporting the methodological assumption that diagnostic imputation accuracy predicts real-world performance.

## Discussion

### Key results

This study presents a practical algorithm selection framework for imputing missing values in numerical datasets, specifically tailored for biomedical research. By systematically ranking imputation methods on the dataset at hand, the approach addresses a common challenge: the lack of dataset-specific guidance in the selection of imputation techniques. Unlike general recommendations or literature-based choices,^5 6 39 53 54^ this method allows for direct, data-driven evaluation and selection.

The results highlight that multivariate imputation methods generally outperform univariate approaches in datasets with interdependent variables, such as concentration-based biomedical data. This supports the use of multivariate algorithms for such data types and provides a practical tool for method selection. However, the tool is designed for datasets with completely random missing values; cases with nonrandom missingness (e.g., values below detection limits) require special handling prior to imputation.

Both univariate (using only the feature itself) and multivariate (using all features) imputation methods were evaluated, with multivariate approaches using all available features in a regression framework (e.g., random forests for regression, not classification), without requiring feature selection, and recognizing that multivariate approaches often perform better, although performance is dataset dependent.^6 7^ A novel z-metric was used to rank algorithm performance, resulting in a clear recommendation tailored to the dataset at hand. Importantly, the method also signals when all tested algorithms perform poorly, alerting researchers to potential risks in subsequent analyses.

### Challenges in psychophysical pain data

Psychophysical pain datasets presented significant challenges for imputation. Here, even the best available methods failed to outperform biased or “poisoned” imputations, suggesting limited structure and high complexity in pain sensitivity data. This observation is consistent with previous findings regarding the low correlation between pain sensitivity measures and the inherent complexity of pain perception.^55–57^ It also highlights the limitations of traditional imputation and clustering methods for such data, suggesting that more advanced techniques, such as neural networks or generative AI approaches,^58–60^ may be required to capture complex data structures.

### Downstream impact on subgroup structures

To demonstrate that inadequate choice of imputation methods can have severe consequences for subsequent data analysis, we investigated both a synthetic and a real lipidomics dataset, which transparently illustrate this point. Specifically, we focused on two key downstream applications, that is, clustering and classification.

#### Clustering example

In the “HEPTA” clustering dataset (dataset #7, see Supplementary materials), no multivariate imputation method outperformed simple univariate approaches, and a poisoned method was recommended, indicating non-imputability (Supplementary Figure 2). Therefore, we reverted to median imputation and performed clustering using the k-means algorithm,^61 62^ followed by alignment of the resulting cluster assignments to the original clustering labels to enable comparison. While median imputation preserved the correct number of clusters, clustering accuracy was reduced due to misclassification (Supplementary Figure 2C). In biomedical contexts, such errors in subgroup identification can have significant implications for research interpretation.^63^ Notably, excluding imputation and conducting clustering only on complete cases preserved perfect cluster assignment. This example highlights that when imputation does not meaningfully improve data quality or downstream analyses, opting to forgo imputation and rely on complete case analysis remains a valid and sometimes preferable strategy, especially when missingness is limited or random.

#### Classification example

In the complete lipidomics dataset (#4b), 18 missing values were imputed using two methods: Amelia repeated imputation, identified as the best-performing approach in the main analyses, and simple median imputation. Random forest classifiers were then trained separately on each imputed dataset to predict diagnostic categories, including depression, bipolar disorder, ADHD, dementia, and controls. Performance was evaluated using out-of-bag (OOB) error rates and confusion matrices. The OOB error rate for the Amelia-imputed data was 70.2 percent, slightly lower than the 73.4 percent observed for the median-imputed data, indicating marginally better, yet still relatively high, classification error. Differences in classification accuracy per class were minor (see Supplementary Figure 3). This relatively high error rate likely reflects the exploratory nature of the present analysis, whereas the original assessments involved more rigorous methodological tuning aimed at improved results.^28^ The primary aim here was to illustrate how the choice of imputation method can meaningfully impact downstream classification outcomes.

### Limitations and scope of the framework

This report does not introduce new imputation algorithms nor does it aim to exhaustively benchmark all existing techniques. For comprehensive overviews of currently available methods, readers are referred to independent reviews such as.^64^ Instead, we propose a comparative framework for selecting among a representative set of established imputation approaches, which can be readily extended to incorporate other methods as needed. It is assumed that conventional preprocessing steps, such as data transformation, outlier removal, and exclusion of invariant variables, are completed prior to applying the framework. The approach is designed to complement, rather than replace, rigorous data preparation.

#### Extensible model‑agnostic design

The proposed framework is model‑agnostic, enabling the evaluation of imputation algorithms from diverse methodological families. The current implementation represents a practical subset of widely used approaches, such as those available in the R computing environment, without claiming completeness (Table 1). To facilitate alternative software implementations, the full procedure is summarized as pseudo‑code (Textbox 1).

The current selection, as implemented in the accompanying R package, comprises a representative spectrum of established techniques across univariate, multivariate, and ensemble categories (Table 1). Univariate methods include simple statistical estimators such as mean, median, mode, and random sampling. Multivariate methods encompass linear regression, predictive mean matching, k‑nearest neighbor algorithms, and multiple‑imputation procedures (for example, those implemented in MICE and Amelia). Ensemble and tree‑based strategies, such as random forest, bagging, and classification‑and‑regression tree (CART) imputations, complement these, covering the major algorithmic families available within the R ecosystem.

Recent advances in missing‑data imputation have produced a diverse array of statistical and machine‑learning approaches. Traditional univariate and multivariate methods, such as mean, median, predictive mean matching, and multiple imputation via chained equations, remain widely employed due to their ease of implementation and interpretability.^19 38 65^ More recently, ensemble methods such as random‑forest and bagging have demonstrated robust performance across heterogeneous numerical datasets.^41 66 67^ Several benchmarking studies have systematically compared imputation strategies, revealing substantial variation in accuracy and bias depending on data structure and missingness mechanism.^65 67–73^ A PubMed search conducted on October 25, 2025, for “comparison of various imputation algorithms for missing data” yielded 179 publications, underscoring the extensive attention this topic has already received. Rather than adding yet another comparative benchmarking effort, the present work proposes an extensible, model‑agnostic framework to identify the most suitable imputation method for the specific dataset under study.

Additional algorithms, such as deep‑learning architectures or domain‑specific procedures, can be integrated using the same structure. Results derived from external software packages may likewise be imported for standardized evaluation, ensuring methodological extensibility. The framework benchmarks all candidate methods and determines those with dataset‑specific optimum performance. While integrating more advanced or emerging models, such as deep learning, may further enhance imputation accuracy, such extensions lie beyond the present scope. Deep‑learning‑based imputations have shown promise for high‑dimensional omics datasets,^74^ and broad comparative assessments of imputation strategies have recently been reported.^73 75^ However, omics data represent only one class of biomedical datasets. In pain research, by contrast, psychophysical, questionnaire‑based, and clinical data exhibit distinct data structures and missingness patterns that critically influence imputation effectiveness.

A key feature of the framework is the inclusion of diagnostic and poisoned imputation strategies. These reference models provide a controlled basis for assessing potential bias and error inflation, enabling an objective evaluation of whether a more complex model genuinely outperforms baseline procedures. If none of the available techniques demonstrate superiority over these diagnostic standards, the framework equally supports omitting imputation and proceeding with complete‑case analysis.

#### Impact of missingness mechanisms

The effectiveness of any imputation approach is closely linked to the mechanism and randomness of missing data. The current framework assumes data are missing completely at random (MCAR), where the likelihood of missingness is independent of both observed and unobserved variables. Departures from MCAR, such as missing at random (MAR), where missingness depends on observed data, or missing not at random (MNAR), where it is related to unobserved values, typically reduce imputation accuracy.^5 76^ Although the present framework is demonstrated under the MCAR assumption, it is readily extendable to MAR and MNAR scenarios.

#### Alternative strategies for evaluating imputation performance

In its current implementation, the framework assesses imputation performance by comparing imputed values to artificially masked true values, providing a direct and interpretable measure of reconstruction error. Alternative evaluation strategies have been suggested, including comparing the distributional similarity between imputed and original data,^77^ as well as prior uses of distribution‑based assessment for downsampling effects.^78^

Beyond primary error metrics such as RMSD (Equation 1), some studies evaluate imputation in terms of downstream analysis performance, such as classification accuracy.^79^ In this study, such downstream assessments were limited to a post hoc analysis of one dataset. However, optimizing imputation solely for one predictive endpoint risks overfitting and can compromise generalizability, as classification is just one of many possible downstream uses. Adapting imputation method selection to dataset characteristics is therefore supported, considering evidence that no single approach is universally optimal.^77^

## Conclusions and future perspectives

This work introduces a flexible, dataset‑specific model-agnostic benchmarking framework for evaluating missing‑value imputation in pain‑related numerical data. By defining reference conditions that include both “poisoned” and “calibrating” methods, the framework enables objective evaluation of imputation accuracy and bias at the dataset level. Its goal is to guide researchers in selecting the most appropriate imputation techniques—or, when justified, to proceed without imputation—based on empirical evidence rather than convention. Unlike fixed pipelines, the framework is designed to evolve, allowing the seamless integration of emerging algorithms and alternative missingness modeling strategies. Applied to complex biomedical datasets, the framework confirmed that multivariate approaches generally outperform univariate ones when meaningful data structure exists, while also delineating cases where reliable imputation is not feasible.^54 80^ The framework thus provides a transparent, evidence‑based tool to support data integrity and analytical reliability in pain research and beyond.^5 81^

## Supplementary Material

SupplementaryMaterials.pdf

## Availability of data and material - (data transparency)

The data used in the experiments in this report are publicly available and referenced accordingly or can be requested from the first author.

## Code availability - (software application or custom code)

An R package, “opImputation,” which provides key functions for imputation, result evaluation, and the generation of main plots, is accessible at https://github.com/JornLotsch/opImputation. The library has been uploaded to the Comprehensive R Archive Network (CRAN; https://cran.r-project.org); publication is pending.
