# Supplementary material for: A model-agnostic framework for dataset-specific selection of missing value imputation methods in pain-related numerical data
Source: Can J Pain. 2026 Jan 29;10(1):2595160. doi: 10.1080/24740527.2025.2595160 (PMC12867358; doi:10.1080/24740527.2025.2595160)

Supplementary information

Description of the synthetic data sets

Three synthetic three-dimensional datasets were constructed to pose specific challenges for imputation and to facilitate transparent evaluation, as their structure is fully defined and mathematically controlled.

Data Set #5 ("TwoLinearXY")

This dataset consists of 100 data points for each of three variables:

$$Var1: x_i = \frac{(i-1)\cdot 10}{n-1} + U_1(n, -0.3, 0.3), where\ i = 1,2,\dots,n$$

$$Var2: y1_i = x_i + U_2(n, -0.3, 0.3)$$

$$Var3: y2_i = \max(x_i) - x_i + U_3(n, -0.3, 0.3)$$

where $U_j(n, a, b)$ denotes n values drawn uniformly from $[a, b]$. The variables form two lines with slopes +1 and –1, creating an “X” pattern. This structure is designed to be imputable by multivariate methods due to the linear relationships among variables.

Data Set #6 ("UniformRandom3VarIndependent")

This dataset also contains 100 data points for each of three variables:

$$Var1: x_i = U_1(n, 0, 10)$$

$$Var2: y1_i = U_2(n, 0, 10)$$

$$Var3: y2_i = U_3(n, 0, 10)$$

All variables are independently and uniformly distributed, representing pure random noise. As such, multivariate imputation methods are not expected to perform better than univariate approaches.

Data Set #7 ("FCPSHepta")

Taken from the Fundamental Clustering and Projection Suite (FCPS) [1], this dataset contains 212 data points distributed among seven well-separated clusters in three-dimensional space. Six clusters each contain 30 points centered on the main axes at a distance of three units from the origin, while the seventh cluster at the origin contains 32 points within a smaller sphere (radius 0.1), resulting in higher density. The data are generated as follows: Points are assigned to clusters and distributed around their respective centers according to spherical coordinates with random radii and angles. The dataset is designed to reflect settings with well-defined, separable clusters and is suitable for benchmarking imputation and clustering methods. The original data, including cluster assignments, are available in the R package "FCPS" (https://cran.r-project.org/package=FCPS [2]).

References

1. Ultsch A, Lötsch J. The Fundamental Clustering and Projection Suite (FCPS): A Dataset Collection to Test the Performance of Clustering and Data Projection Algorithms. Data 2020;5(1):13.

2. Thrun M, Stier Q. Fundamental clustering algorithms suite. SoftwareX 2021;13:100642-.

3. R: A Language and Environment for Statistical Computing [computer program]. Vienna, Austria2021.

4. Olsen LR, Zachariae HB. cvms: Cross-Validation for Model Selection. 2023.

Supplementary Textbox

Supplementary Textbox 1: Demonstration of installation and example usage of the "opImputation" R package. The code snippet illustrates the installation of the package (either from GitHub or from CRAN) and the execution of its main imputation framework using the built-in iris dataset as a standardized demonstration case. The example showcases the automated selection and application of optimal data imputation methods implemented in "opImputation". For further details and advanced options, refer to the package documentation and R help files.

```r
# Install and load the devtools package if not already installed
if (!requireNamespace("devtools", quietly = TRUE)) install.packages("devtools")

# Install opImputation from GitHub (development version)
devtools::install_github("JornLotsch/opImputation")

# Load the package
library(opImputation)

# When the package is available on CRAN, install with:
install.packages("opImputation")

```

```r
# Example: Compare different imputation methods using the iris dataset

# Load example data (only numeric columns)
data_iris <- iris[, 1:4]

# Introduce approximately 5% random missing values per column
set.seed(42)
for (i in 1:4) {
  # Randomly make 5% of the entries in each column missing
  data_iris[sample(1:nrow(data_iris), 0.05 * nrow(data_iris)), i] <- NA
}

# Step 1: Compare several imputation methods
results <- compare_imputation_methods(
  data = data_iris,
  imputation_methods = c("mean", "median", "knn5", "rf_missForest"),  # supported methods
```

```
  n_iterations = 10,            # number of test iterations
  imputation_repetitions = 10, # repetitions for stochastic methods
  seed = 42                    # reproducibility
)

# Step 2: Access the imputed dataset from the best performing method
imputed_data <- results$imputed_data

```

Supplementary Table

Supplementary Table 1: Sample textual output of the automatic imputation selecting R tool. This table presents the performance scores of various imputation methods as evaluated by the automatic imputation selection tool. Each row corresponds to an imputation method and includes the following columns: abc_score (Δz): The calculated score indicating the method's performance, where higher values signify better imputation quality. abc_category: The categorical ranking assigned based on the abc_score, indicating the relative effectiveness of the methods. method: The name of the imputation method used.

abc_score (Δz)	abc_category	method
**36.5755**	A	plusminus
**21.888**	A	cart_repeated
**17.5513**	A	pmm_repeated
**16.875**	A	rf_mice_repeated
**16.081**	A	miceRanger
**15.3061**	A	miceRanger_repeated
**9.7959**	A	cart
**9.0947**	B	pmm
**7.2345**	B	rf_missForest
**7.0602**	B	amelia_repeated
**4.843**	B	miImp
**4.843**	B	rf_mice
**3.2741**	C	plus
**3.2153**	C	rf_missForest_repeated
**1.9199**	C	amelia
**1.4161**	C	knn3
**...**	C	...

Supplementary Figures

Supplementary Figure 1: Results of imputation analysis on two synthetic data sets, the first of which, "Two linear xy data sets forming an X" (Data set #5), contains three linearly interdependent variables, while the second, "UniformRandom3VarIndependent" (Data Set #6), presents imputation challenges because the value of one variable is not easily predictable from the values of the other variables because the variables contain independent random noise. The panels show, from top to bottom, the color-coded cABC category ranking results of the imputation methods (panels **A and D**), the mean absolute standardized errors, zΔ, obtained with different imputation models using imputed "diagnostic" missing values (panels **B and E**), and the imputation errors per variable (panels **C and F**). For more details on the presentation of the plots, see Figure 1. An "x" denotes an early termination of the imputation with an error message, which is occasionally observed.

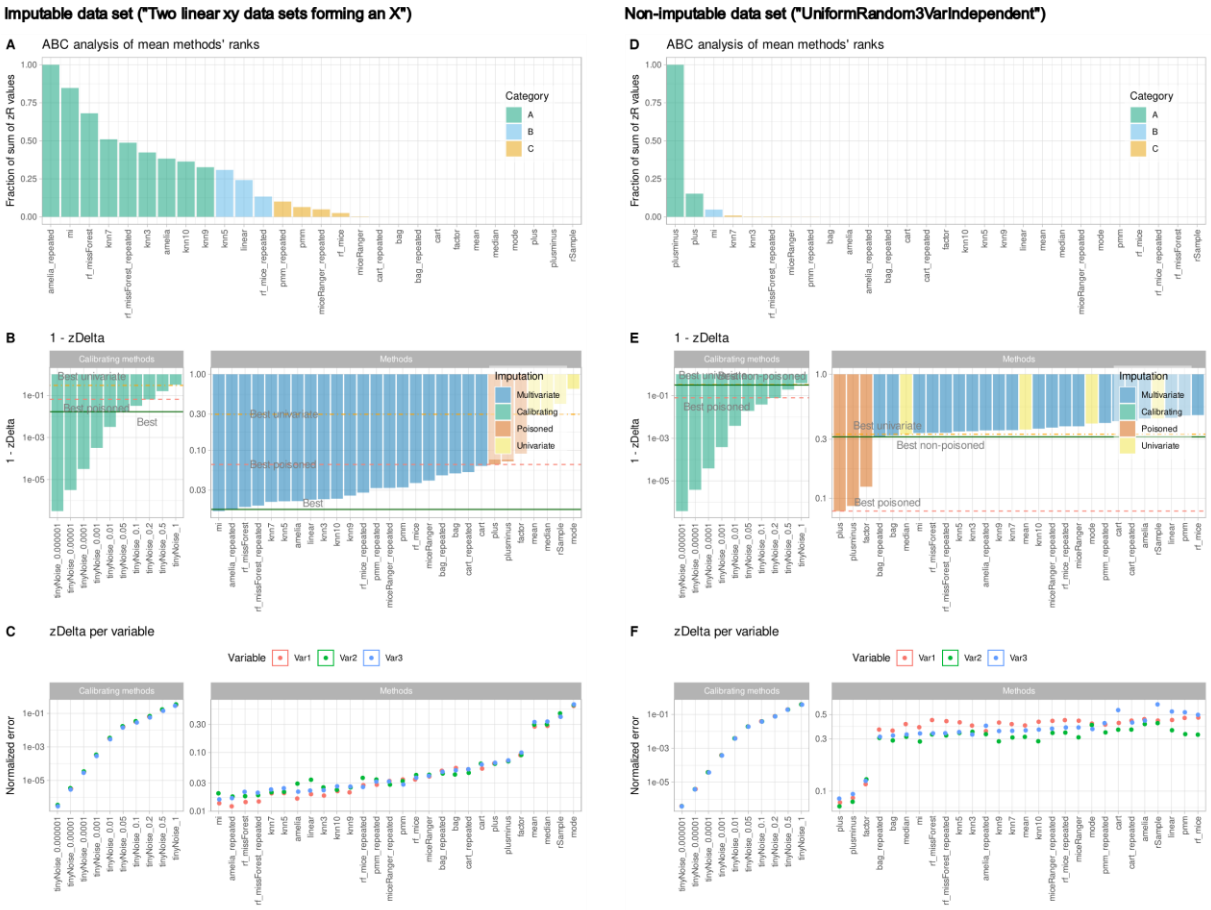

Supplementary Figure 2: Consequences of resorting to median imputation when multivariate imputation proves ineffective in yielding more accurate results. The synthetic "Hepta" data set #7 [1] comprising n = 212 data points evenly distributed across k = 7 classes within well-defined and well-separated spherical clusters. **A**: Bar graph of standardized mean ranks, color-coded by cABC categories of the imputation algorithm, and scaled to fit ABC curves. ABC sets A, B, and C represent best, next best, and discouraged models. The superimposed ABC curve (black line) shows from bottom to top the increasing fraction of the total sum of z-transformed mean ranks of the imputation models, and from left to right the increasing fraction of the total ranks. Boundaries are indicated by dotted or dashed lines. **B:** Bar charts showing the mean absolute standardized errors, zΔ, obtained with different imputation models using inserted "diagnostic" missing values. The color code represents univariate (yellow), multivariate (blue), or pseudo ("poisoned", orange) imputation models. Horizontal lines mark the best models within each model type. Here, the best “non-poisoned” model overall was identical with the best univariate imputation model. An "x" denotes an early termination of the imputation with an error message, which is occasionally observed. The left panel shows the zΔ values obtained with the calibrated imputation models. **C:** While median imputation restored the k = 7 clusters, a closer inspection revealed that the central cluster was inaccurately composed of former members of neighboring clusters. On the contrary, clustering the dataset with missing values provided the correct clusters, albeit in fewer instances. **D:** Bar graph of the cluster accuracy obtained when utilizing imputed datasets with various imputation models.

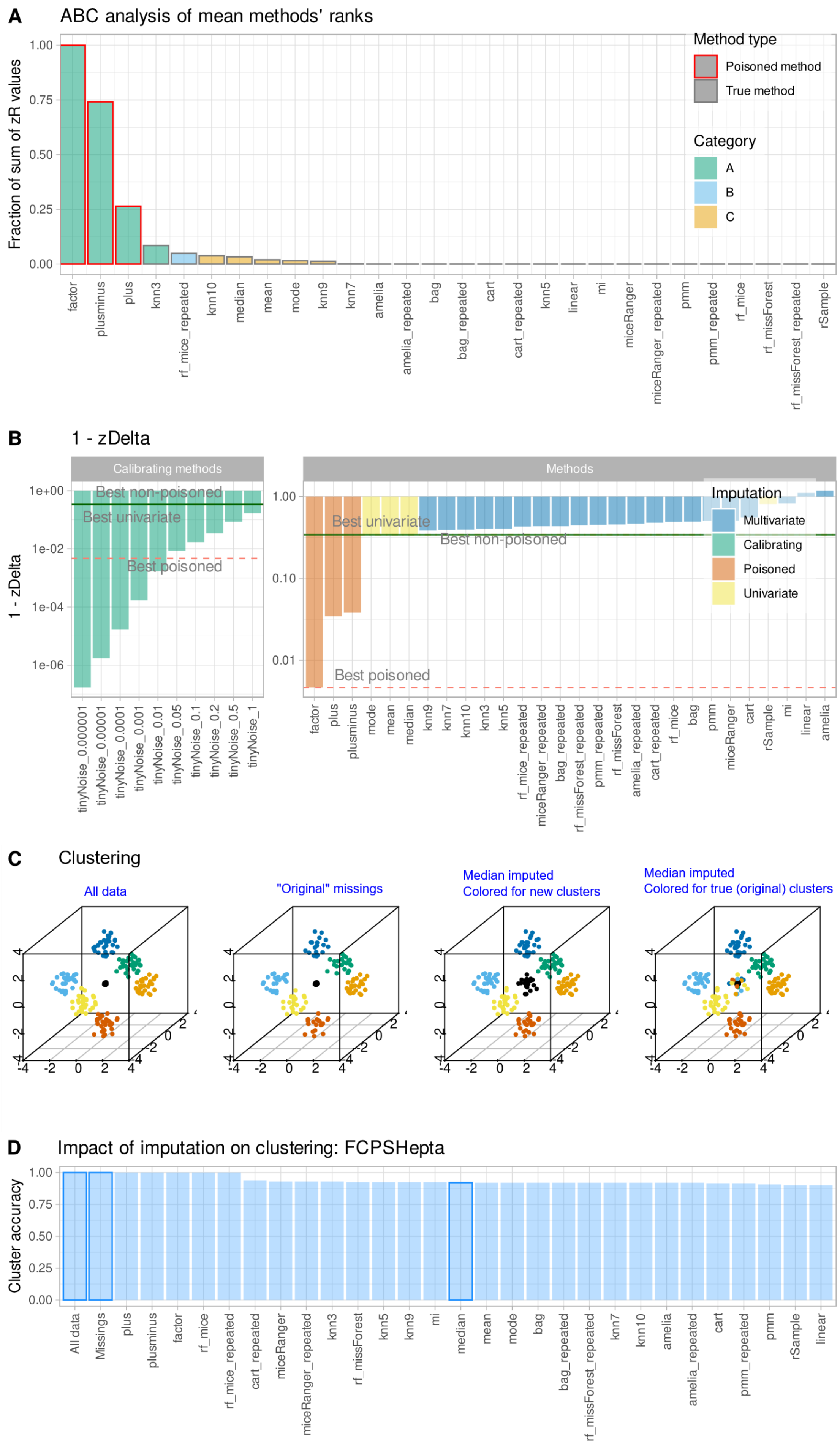
A
ABC analysis of mean methods' ranks
Fraction of sum of zR values
1.00
0.75
0.50
0.25
0.00
Method type
Poisoned method
True method
Category
A
B
C
factor
plusminus
plus
knn3
rf_mice_repeated
knn10
median
mean
mode
knn9
knn7
amelia
amelia_repeated
bag
bag_repeated
cart
cart_repeated
knn5
linear
mi
miceRanger
miceRanger_repeated
pmm
pmm_repeated
rf_mice
rf_missForest
rf_missForest_repeated
rSample
B
1 - zDelta
Calibrating methods
Methods
1 - zDelta
1e+00
1e-02
1e-04
1e-06
1.00
0.10
0.01
Best non-poisoned
Best univariate
Best poisoned
Imputation
Multivariate
Calibrating
Poisoned
Univariate
tinyNoise_0.000001
tinyNoise_0.00001
tinyNoise_0.0001
tinyNoise_0.001
tinyNoise_0.01
tinyNoise_0.05
tinyNoise_0.1
tinyNoise_0.2
tinyNoise_0.5
tinyNoise_1
factor
plus
plusminus
mode
mean
median
knn9
knn7
knn10
knn3
knn5
rf_mice_repeated
miceRanger_repeated
bag_repeated
rf_missForest_repeated
pmm_repeated
rf_missForest
amelia_repeated
cart_repeated
rf_mice
bag
pmm
miceRanger
cart
rSample
mi
linear
amelia
C
Clustering
All data
"Original" missings
Median imputed
Colored for new clusters
Median imputed
Colored for true (original) clusters
D
Impact of imputation on clustering: FCPSHepta
Cluster accuracy
1.00
0.75
0.50
0.25
0.00
All data
Missings
plus
plusminus
factor
rf_mice
rf_mice_repeated
cart_repeated
miceRanger
miceRanger_repeated
knn3
rf_missForest
knn5
knn9
mi
median
mean
mode
bag
bag_repeated
rf_missForest_repeated
knn7
knn10
amelia
amelia_repeated
cart
pmm_repeated
pmm
rSample
linear

Supplementary Figure 3: Comparison of confusion matrices from Random Forest models trained on lipidomics data with two different missing value imputation methods: MissForest (left) and Median imputation (right). Each matrix shows counts of predicted versus actual class labels (A, B, C, D, E). In the center of each tile, the normalized number (total percentage) of cases assigned to each class is displayed; below it is the number of cases with an additional indication of the number of cases per true class. The column percentages are shown at the bottom, and the row percentages are shown to the right of each tile. The color intensity in each cell corresponds to the number of samples with that combination of true and predicted labels, with darker colors representing higher counts. Diagonal cells represent correctly classified samples, while off-diagonal cells indicate misclassifications. The "Sum" rows and columns show the total count per predicted and actual class, providing insight into the class distribution and prediction tendencies. The figure was generated using R software (version 4.3.3 for Linux; https://CRAN.R-project.org/ [3]) and the "cvms" library (https://cran.r-project.org/package=cvms [4]).

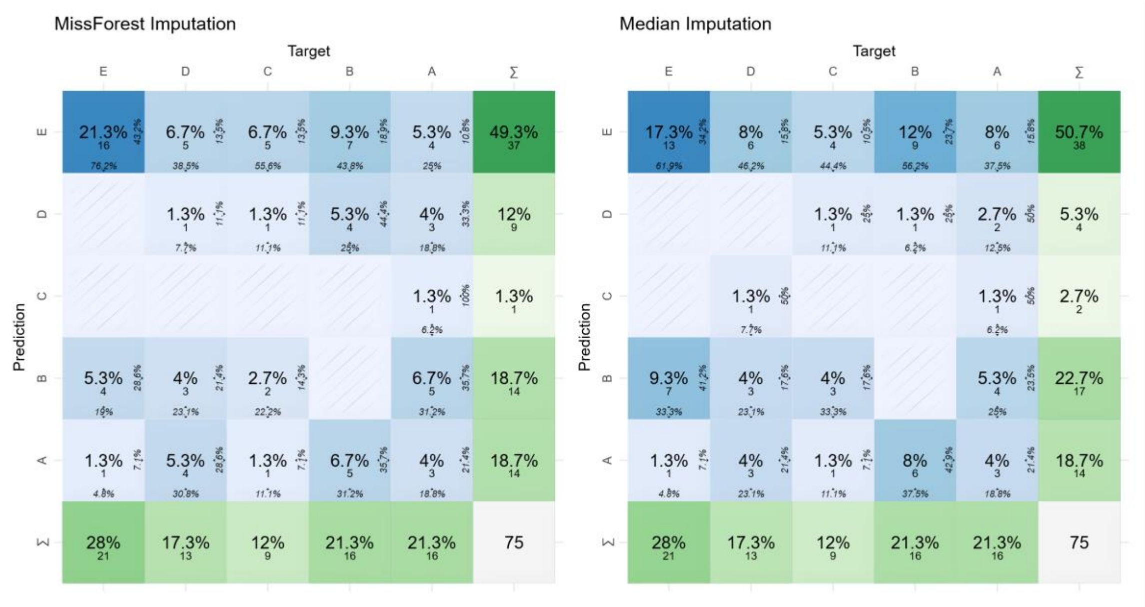

Supplement: SupplementaryMaterials.pdf [file UCJP_A_2595160_SM3084.pdf]
